# Analysis of Human Faecal Host Proteins: Responsiveness to 10-Week Dietary Intervention Modifying Dietary Protein Intake in Elderly Males

**DOI:** 10.3389/fnut.2020.595905

**Published:** 2021-01-13

**Authors:** Jessica L. Gathercole, Anita J. Grosvenor, Erin Lee, Ancy Thomas, Cameron J. Mitchell, Nina Zeng, Randall F. D'Souza, Farha Ramzan, Pankaja Sharma, Scott O. Knowles, Nicole C. Roy, Anders Sjödin, Karl-Heinz Wagner, Amber M. Milan, Sarah M. Mitchell, David Cameron-Smith

**Affiliations:** ^1^Proteins and Metabolites Team, AgResearch, Lincoln, Christchurch, New Zealand; ^2^School of Kinesiology, University of British Columbia, Vancouver, BC, Canada; ^3^Liggins Institute, University of Auckland, Auckland, New Zealand; ^4^Discipline of Nutrition, University of Auckland, Auckland, New Zealand; ^5^Food, Nutrition, and Health Team, AgResearch, Auckland University, Auckland, New Zealand; ^6^Department of Nutrition, University of Otago, Dunedin, New Zealand; ^7^Riddet Institute, Massey University, Palmerston North, New Zealand; ^8^High-Value Nutrition National Science Challenge, Auckland, New Zealand; ^9^Department of Nutrition, Exercise, and Sports, Copenhagen University, Copenhagen, Denmark; ^10^Department of Nutritional Sciences and Research Platform Active Ageing, University of Vienna, Vienna, Austria; ^11^Agency for Science, Technology, and Research, Singapore Institute for Clinical Sciences, Singapore, Singapore

**Keywords:** faeces, dietary protein, host proteins, gastrointestinal health, proteomics

## Abstract

Faecal proteomics targeting biomarkers of immunity and inflammation have demonstrated clinical application for the identification of changes in gastrointestinal function. However, there are limited comprehensive analyses of the host faecal proteome and how it may be influenced by dietary factors. To examine this, the *Homo sapiens* post-diet proteome of older males was analysed at the completion of a 10-week dietary intervention, either meeting the minimum dietary protein recommendations (RDA; *n* = 9) or twice the recommended dietary allowance (2RDA, *n* = 10). The host faecal proteome differed markedly between individuals, with only a small subset of proteins present in ≥ 60% of subjects (14 and 44 proteins, RDA and 2RDA, respectively, with only 7 common to both groups). No differences were observed between the diet groups on the profiles of host faecal proteins. Faecal proteins were detected from a wide range of protein classes, with high inter-individual variation and absence of obvious impact in response to diets with markedly different protein intake. This suggests that well-matched whole food diets with two-fold variation in protein intake maintained for 10 weeks have minimal impact on human faecal host proteins.

## Introduction

The gastrointestinal tract (GIT) coordinates the complex tasks of digestion and nutrient absorption (1, 2). Beyond these functions, it is increasingly understood that the GIT interacts symbiotically with the resident microbiome population (3). Given the inherent complexity of both nutrient digestion and sustaining the symbiotic gut microbiome, the GIT requires the coordinated functioning of a large network of immune, secretory and neural cells and systems that exhibit specialisation and coordinated functionality along its length (4). This entire GIT system achieves the coordination required to digest and/or eliminate a staggering diversity of ingested compounds, including potentially pathogenic microorganisms. For the majority of individuals this occurs in the absence of discomfort or illness, yet estimates suggest that up to 35% of people over 65 years old suffer from a chronic gastrointestinal disease in the USA and 25% of women in the Zurich Cohort study (5). This includes a diverse array of functional gastrointestinal disorders (FGIDs), including functional dyspepsia (FD) and irritable bowel syndrome (IBS), that are characterised on the basis of differing combinations of chronic or acute gastrointestinal symptoms. Functional gastrointestinal disorders are defined by their lack of explanatory GIT structural or biochemical abnormalities that account for this symptomology. Therefore, there is an ongoing requirement to gain insight into GIT function in healthy individuals and the mechanisms which may underpin FGID development.

To date, high throughput methods to comprehensively profile the genes and proteins from the microbiota have been applied to the analysis of the biological functioning of the human GIT. These techniques have been used to comprehensively describe the taxonomy and functional attributes of the microbiome population (6, 7). Fewer studies have addressed the inherent expression and abundance of self-derived genetic material or proteins. This is important, as host-derived inflammatory and heightened immune response are a hallmark feature of GIT diseases and in situations of intestinal dysbiosis. This is evident in the analysis of biomarkers, including calprotectin and lactoferrin (8) in faecal matter. Experimental studies have identified a widening list of possible protein candidate for disease associations, including calprotectin, pyruvate kinase, myeloperoxidase and matrix metalloproteinase protein family members (8–10). Yet whilst these markers have potential as disease biomarkers (8), these discrete proteins provide little insight into the complexity of the disturbances in the GIT, providing very limited understanding into the altered functioning of the complex cellular systems underpin the disease aetiology and pathobiology.

Current proteomic techniques are capable of measuring thousands of proteins. However, significant challenges remain for proteomic application in human faecal samples. The faecal proteome is inherently complex because it contains various groupings of proteins that are, respectively, derived from either the host, the microbiome and proteome remnants from the ingested food (9, 11, 12). Further, data dependent analysis of peptides in mass spectrometry is frequently limited to the most abundant peptides. Dynamic exclusion is then used to prevent the same peptide being analysed twice over the chromatographic peak (13, 14). A more complete proteome can be obtained using fractionation prior to LC, which reduces the complexity of each LC separation and allows for more peptides to be fragmented and thus identified when comparing to analysing the whole sample in one run (15). These fractions can be combined prior to searching to identify all compounds at once. This strategy is applied in many proteomic analysis (e.g., shotgun proteomics which separated peptides by their ionic strength followed by their hydrophobicity prior to MS/MS analysis) (16). Different extraction, preparation and fractionation procedures for faecal proteomics have been used which are more beneficial to different parts of the proteome. For the current study SDS extraction was used to improve protein extractability from the samples (17).

Of particular interest in the regulation of the GIT host proteome is the impact of dietary protein. High protein diets are the subject of considerable interest, given the proposed benefits for appetite regulation, cardiovascular health, glucose homeostasis, body condition and weight loss (18). Studies on elderly people suggest a potential requirement for a greater daily protein or amino acid intake to aid in sustaining skeletal muscle mass and function (19, 20). Yet, protein digestion is likely to have significant impact on the GIT. Experimental rodent studies demonstrate that alteration in protein diet affected small intestinal jejunal and goblet cell function, with altered protein expression and mucus secretions (21–24). Similarly, clinical analysis of the impact of isocaloric substitution of maltodextrin for protein (casein or soy protein) identifies marked changes in the mucosal gene transcriptome obtained from rectal biopsies (25). The current study used an untargeted discovery proteomics which allows for all proteins in humans to be identified instead of a sub-set of proteins in animals which were done in previous studies. This process was used to identify the faecal host proteome classes in a cohort of older males who were fed on either the RDA diet (0.8 g/kg/day), which included the recommended dietary allowance for 10 weeks or 2RDA, containing double the recommended dietary allowance (1.6 g/kg/day), as previously described (26). We understood that the faecal host proteome would include proteins secreted into the gastrointestinal tract, including enzymes, mucus proteins, secretory, immune proteins and shredded cells (4). We further hypothesised the diet can affect the presence of human (self) proteins after the 10-week diet. Although food and microbiota protein fragments were observed the discussion on these is part of other work.

## Experimental

### Diet and Sample Collection

Nineteen healthy older men aged 70 years and above with BMI (kg/m^2^) between 18 and 35 were recruited from the local community to participate in the study. The RDA group had an average age of 75.2 ± 4.5 s.d. years and an average BMI of 27.3 ± 4.7 s.d. kg/m^2^. The 2RDA group had an average age of 73.8 ± 3.5 s.d. years and an average BMI of 28.3 ± 3.3 s.d. kg/m^2^. All were non-smokers and not consuming dietary supplements for at least 1 month prior to participating in this trial. Potential participants were excluded if they adhered to restricted diet practises, including vegetarians or those with self-reported food allergies or intolerances (e.g., nuts, fish, dairy). Further exclusion was applied to those with a prior history of digestive or cardio-metabolic disease.

#### Experimental Design

The design of this trial has been previously described (26). Participants were randomised into two groups, where 9 participants received a controlled diet of 0.8 g protein/kg/d (RDA) and 10 participants received a controlled diet of 1.6 g protein/kg/d (2RDA) for 10 weeks. Protein equal to twice the recommended dietary allowance was chosen because evidence showed that protein at this level is absorbed well before the large intestine (26). All meals consumed by the participants were provided by the investigators. The percentage of energy derived from fat was 28–31%, from proteins it was 11.7% for RDA and 20.6% for 2RDA. The remaining energy was made up from carbohydrates. All diets adhered to Eating and Activity Guidelines for New Zealand and met recommendations for intake of fruit and vegetables (27). All participants completed dietary records to ensure all food provided was consumed, and food selection was adjusted according to participants' preferences to maintain high compliance. Any non-study food consumed was also recorded. The energy content of the intervention diet was individually calculated to match participants' estimated energy needs based on the Harris-Benedict equation and adjusted for physical activity, which was assessed by wrist-worn accelerometers (Fitbit Charge HR). The estimated energy needs were calculated before the intervention and adjusted fortnightly based on participant satiety and weight maintenance to ensure participants consumed adequate protein relative to energy intake. During the intervention participants were instructed to maintain their normal lifestyle, and prepared meals were delivered to their homes. All testing was conducted at the University of Auckland Nutrition and Mobility Clinic.

#### Sample Collection and Storage

Faecal samples were collected during the 10th week of the study, post-intervention. Briefly, participants were provided with a sample collection kit and instructions for collection at home. Once collected, samples were couriered to the Liggins Institute (Auckland, New Zealand) on ice within 3 h and stored immediately at −80°C. For proteomic analysis, 1–2 g was aliquoted from the frozen sample and shipped on ice for proteomic analysis. The faeces were separated into particulate matter and supernatant based on previously published protocols (17, 28). Guanidine hydrochloride was added to denature proteins and limit the activity of bacterial proteins.

### Preparation for Proteomics

**Figure 1:**
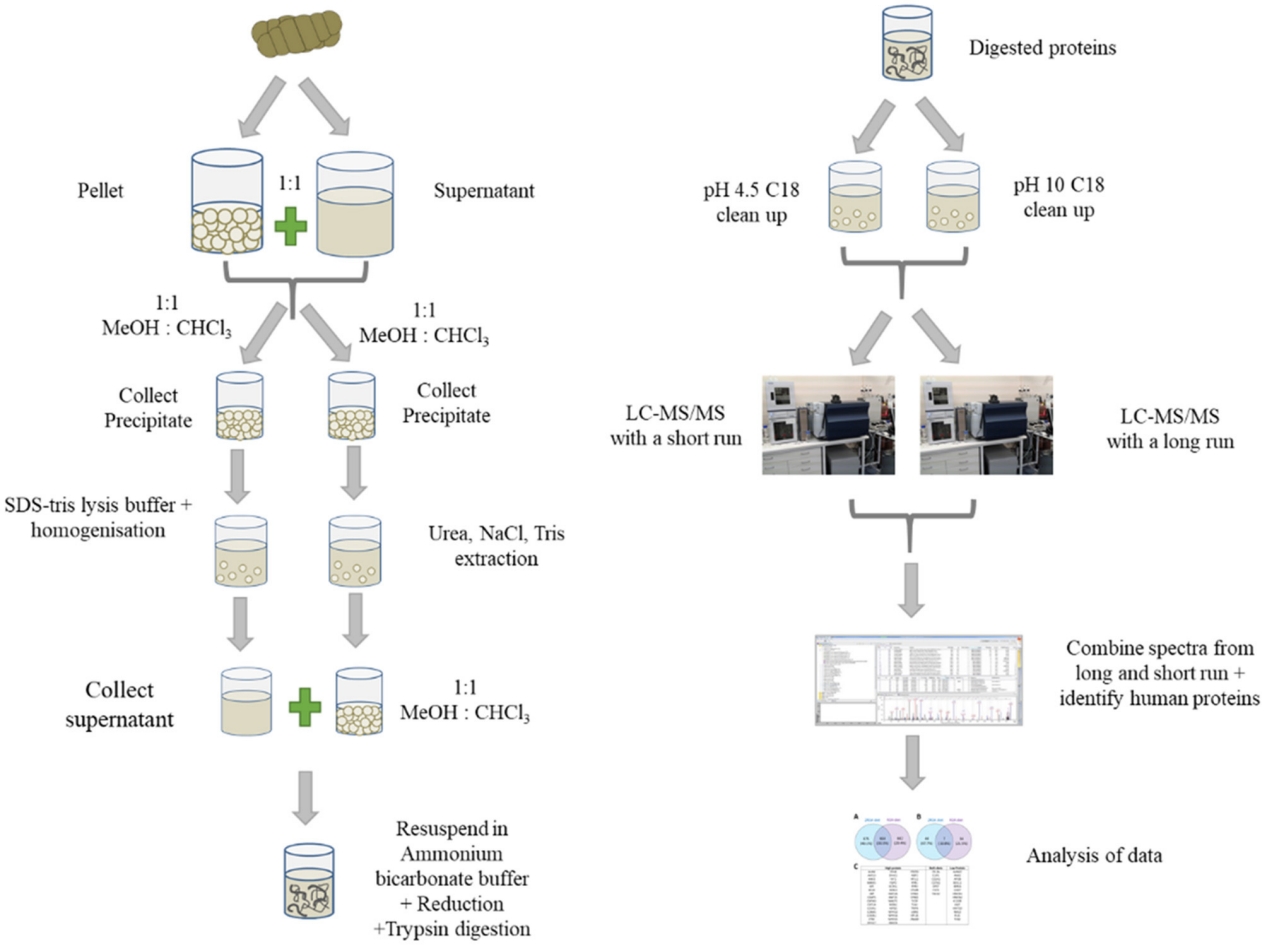
Schematic showing the preparation of faecal samples for proteomics, fractionation, analysis on LC-MS/MS and data analysis.

A schematic showing the preparation and analysis of samples for proteomics is shown in Figure 1. The pellet and supernatant (see section Sample Collection and Storage) were combined 1:1. Methanol—chloroform was used to extract proteins and remove guanidine hydrochloride as described previously (29). The precipitated protein was resuspended in two extraction buffers—lysis buffer and urea-tris buffer. For the lysis buffer, the precipitate was resuspended in buffer containing (4% w/v sodium deoxycholate, 50 mM tris(hydroxymethyl)aminomethane, pH 8.0 using hydrochloric acid). This homogenate was heated to 95°C for 10 min, followed by homogenising in a ground glass tube for 1 min with an electric hand drill (Firebreak 50 Hz P/N 0–2,809 RPM). For the urea buffer, the precipitated peptides were resuspended in urea- tris buffer [8 M urea, 100 mM NaCl and 25 mM tris(hydroxymethyl)aminomethane]. The mixture was pipetted up and down and then vortexed at room temperature overnight. All extracts from both methods were then centrifuged at 14 100 × *g* for 10 min, followed by the collection of the clear supernatants. Methanol—chloroform extraction was done on urea- tris buffer extraction samples to remove urea and isolate the proteins as described in Gathercole et al. (30).

For each sample, the supernatants from both extracts was combined and then dried down using a speed vacuum concentrator. The proteins were resuspended in 50 mM ammonium bicarbonate, pH 8. The proteins were reduced by addition of 1 mmol of tris(2-carboxyethyl)phosphine heated at 56°C for 45 min followed by alkylation with 3 mmol of iodoacetamide, incubated at room temperature in the dark. The proteins were digested overnight at 37°C after the addition of trypsin (~1 μg of trypsin to 133 μg of protein) and final concentration of 10% v/v acetonitrile. To cease the digestion and precipitate sodium deoxycholate, formic acid was added to a concentration of 1% v/v. After centrifugation, the clear supernatant was split in two, dried and purified using Empore C18 disks in both acidic and basic conditions. For acidic conditions, the peptides were resuspended in 0.1% v/v formic acid (pH 4.5). For basic (pH 10) conditions, the peptides were resuspended in 10 mM ammonium formate (pH 10). Three conditioned Empore discs were incubated in these solutions for 3 h. The Empore discs were eluted with vortexing with 75% v/v acetonitrile for 1 h. After removal of the discs, the peptide solutions were dried in a speed vacuum concentrator. The dried peptides were resuspended in 100 μl of 0.1% v/v formic acid. The two C18 elutions were combined 1:1 for each sample prior to LC-MS/MS analysis.

### LC-MS-MS Analysis

The combined C18 elutions for each sample were run in random order with two different separation methods and the mgf (spectra) files were combined prior to protein searching. Samples were injected (5 μl) onto a ProntoSIL C18AQ Nano trap column (5 μm, 200Å) at a flow rate of 5 μl/min. The trap column was then switched in-line with the ProntoSIL C18AQ (100 μm ID × 150 mm, 3 μm, 200 Å) analytical column on a NanoAdvance LC (Bruker Daltonics) in nanoflow mode. After separation the analytes were injected in to the CaptiveSpray followed by an ion trap mass spectrometer (Amazon, Bruker Daltonics). A Nanobooster (Bruker Daltonics) was attached to insert acetonitrile into the captive spray to improve sensitivity. Separation method one was run at 50°C and involved starting with 98% solvent A (0.1% formic acid in water), increasing to 5% B (0.1% formic acid in acetonitrile) at 5 min followed by increasing to 25% B at 65 min then 35% B at 75 min. The column was then cleaned by increasing to 95% B at 80 min, holding for 5 min and then re-equilibrating at 2% B until the end of the 90 min run. The flow rate was set to 800 nL/min. Separation method two was run with a column temperature of 60°C and involved starting with 98% solvent A (0.1% formic acid in water), and 2% solvent B (0.1% formic acid in acetonitrile), increasing to 45% B at 60 min. The column was then cleaned by increasing to 95% B at 62 min, holding for 3 min and then re-equilibrating at 2% B until the end of the 70 min run. The flow rate was set to 400 nl/min. For both separation methods, the MS mode was run with CID positive mode looking for compounds between 350 and 1,200 m/z. MS/MS was done on 10 precursors at a time. Compounds analysed by MS/MS were excluded after 1 spectra for 0.20 min unless the intensity increased by at least 5-fold.

The mass spectrometry proteomics data have been deposited to the ProteomeXchange Consortium via the PRIDE (31) partner repository with the dataset identifier PXD021424.

### Protein Identification

The MS data was exported to ProteinScape (Version 3.1.0 348; Bruker Daltonics). The file for both separation methods was combined into one for each sample. Protein searches were conducted using Mascot Server v 2.5.1 (Matrix Science, UK). Spectra were searched against the Swissprot *Homo sapiens* database. Semitrypsin was selected as the enzyme specificity allowing up to two missed cleavages. The MS error tolerance was set to 0.3 Da and the MS/MS error tolerance was set to 0.8 Da. Peptide and protein Mascot threshold scores were set to 20 and 80, respectively. Instrument specificity was set to ESI-TRAP. The modifications included were carbamidomethyl (C) as fixed and oxidation (M), ammonia loss (N-term-C), sodium (DE) and deamidation (NQ) as variable modifications. Protein identifications required at least one unique peptide identification from the list of identified peptides for that protein.

### Protein Group Identification

To identify protein classes and function, protein identifications were converted from the UNIPROT accessions to gene names using the UNIPROT identification conversion API available at (https://www.uniprot.org/help/api_idmapping). The resulting UNIPROT identifications were then used to query the Human Gene Nomenclature Committee (HUGO) database for additional annotations, including the PANTHER gene annotations (http://www.pantherdb.org version 14.0). PANTHER terms were associated with the related accessions using the R-package PANTHER.db.

## Results and Discussion

The human or host proteome isolated from faecal samples may be an insightful strategy to profile the adaptive regulation of the complex physiological processes associated with nutrient digestion and gut microbiome homeostasis. We hypothesised that the faecal host proteome would include proteins secreted into the gastrointestinal tract, including enzymes, mucus proteins, secretory immune proteins and potentially proteins from epithelial cells dislodged along the GIT (4). We further hypothesised that significantly different diets would change the faecal self-proteome. In the current study LC-MS analysis was undertaken on stool samples isolated after healthy elderly male volunteers had consumed one of two diets differing in total protein content (26). The RDA diet satisfied the minimum WHO dietary protein guidelines and the 2RDA diet provided double this protein amount, with compensatory changes in carbohydrate intake, to maintain energy-balance.

Although in this manuscript we looked into the self-proteome, the samples also included proteins from the microbiome and fragments of dietary proteins. As mass spectrometry cannot select only the human peptides in the LC-MS/MS run, fractionation was performed on the samples prior to LC separation to increase peptide and thus protein identification. Fractionation allows for more peptides to be fragmented when using data-dependent analysis with MS. To limit the peptide identifications to humans, spectra were compared to human peptides in the Swissprot database and a unique peptide was required for each protein identification. Although proteins from food and the microbiota would be present, the searches were restricted to the human proteome database in accordance with the aim of this work. A list of the proteins identified for each sample can be found in the Supplementary Material.

**Figure 2:**
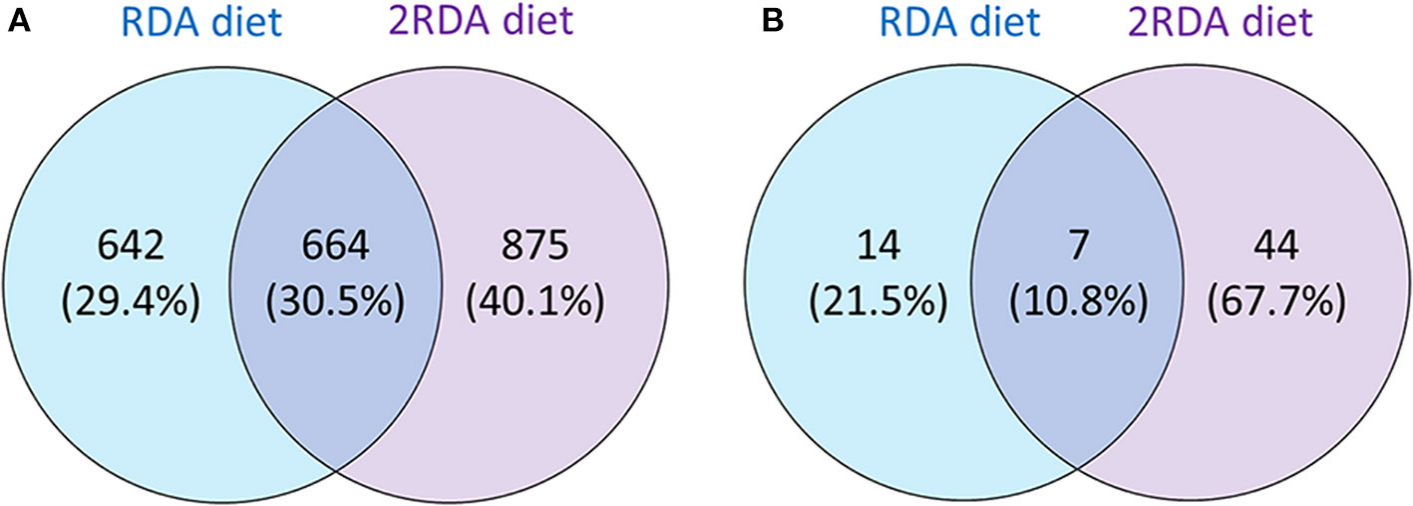
Showing the number of all identified proteins unique to each diet or common to each diet **(A)**; and proteins present in at least 60% of one diet group (*n* = 6 for both diet groups) observed in either one or both diet groups **(B)**.

While investigating differences between the two diets, the proteins identified in each group were examined for differences using a Venn diagram (see Figure 2). The first Venn diagram (Figure 2A) showed the complete list of proteins identified in the 19 samples. Unique proteins were found in the RDA and the 2RDA diets but 30.5% of the proteins (664) were found in both diets. A higher number of host human proteins were identified from the 2RDA diet samples. An average of 233 ± 11 s.d. proteins and 575 ± 28 s.d. peptides were identified in the RDA protein diet samples and 266 ± 14 proteins and 637 ± 38 peptides were identified in each of the 2RDA diet samples. There was no significant difference in the number of identifications between the two diets (*p* = 0.07 for proteins and *p* = 0.21 for peptides). There were, however, large differences observed between individuals. Overall fragments from 2181 different host-proteins were identified in the 19 samples and 76% of these proteins were identified in only two or fewer samples. Thus, there was very limited overlap between individuals for the majority of identified proteins. The protein identified most consistently across all samples was chymotrypsin-like elastase family member 3A (Uniprot ID CEL3A). CEL3A was present in all of the 2RDA diet samples and absent in just 1 sample from the RDA protein group. CEL3A, also known as Elastase 3A, is a serine protease that is secreted by the pancreas (32).

To observe differences in proteins that are more consistently specific to each diet, the second Venn diagram (Figure 2B) showed the proteins found in at least 60% of the samples with the same diet (proteins listed in Table 1). In the RDA diet, 14 proteins were unique to the proteins found in ≥ 60% of the samples and 44 proteins were found only in the 2RDA diet. Only 7 proteins were found in 60% of the samples for both diets). None of the proteins were observed in all of the 19 samples. To see if any of these common proteins were unique to either diet, we checked the proteins against the full list for the other diet. All of the 51 proteins in the 2RDA diet were found in at least one sample in the RDA diet.

**Table 1:** Frequency of host proteins found in at least 60% (*n* = 6) of at least one of the diet groups and corresponds to the Venn diagram in Figure 2B.

**Accession**	**Protein name**	**RDA**	**2RDA**
**Found in** **≥** **60% of RDA and 2RDA samples**			
CEL3A	Chymotrypsin-like elastase family member 3A	8	10
CO7A1	Collagen alpha-1(VII) chain	6	9
CLIP1	CAP-Gly domain-containing linker protein 1	7	8
FAT3	Protocadherin Fat 3	6	7
TACC2	Transforming acidic coiled-coil-containing protein 2	6	7
CO2A1	Collagen alpha-1(II) chain	6	6
DYST	Dystonin	6	6
**Found in** **≥** **60% of RDA and** **≤** **60% of 2RDA samples**			
BD1L1	Biorientation of chromosomes in cell division protein 1-like 1	7	5
BIRC6	Baculoviral IAP repeat-containing protein 6	7	5
KMT2D	Histone-lysine N-methyltransferase 2D	7	5
K1109	Uncharacterized protein KIAA1109	7	4
APOB	Apolipoprotein B-100	6	5
HMCN1	Hemicentin-1	6	5
TLN2	Talin-2	6	5
ANK2	Ankyrin-2	6	4
HMCN2	Hemicentin-2	6	4
NAV2	Neuron navigator 2	6	4
KI67	Antigen KI-67	6	3
PLEC	Plectin	6	3
CHD7	Chromodomain-helicase-DNA-binding protein 7	6	2
**Found in** **≥** **60% of 2RDA and** **≤** **60% of RDA samples**			
FSIP2	Fibrous sheath-interacting protein 2	5	9
AHNK	Neuroblast differentiation-associated protein AHNAK	4	9
VP13C	Vacuolar protein sorting-associated protein 13C	3	9
CTRC	Chymotrypsin-C	2	9
RYR1	Ryanodine receptor 1	2	9
BCL9	B-cell CLL/lymphoma 9 protein	4	8
MACF1	Microtubule-actin cross-linking factor 1, isoforms 1/2/3/5	3	8
DYHC1	Cytoplasmic dynein 1 heavy chain 1	1	8
HERC2	E3 ubiquitin-protein ligase HERC2	5	7
KMT2A	Histone-lysine N-methyltransferase 2A	5	7
MDN1	Midasin	5	7
RYR3	Ryanodine receptor 3	4	7
SYNE2	Nesprin-2	4	7
TLN1	Talin-1	4	7
UBR4	E3 ubiquitin-protein ligase UBR4	4	7
ZN469	Zinc finger protein 469	4	7
CNTLN	Centlein	3	7
OBSCN	Obscurin	3	7
STAR9	StAR-related lipid transfer protein 9	3	7
DYH8	Dynein heavy chain 8, axonemal	2	7
SYNE1	Nesprin-1	2	7
ANK3	Ankyrin-3	5	6
CO3A1	Collagen alpha-1(III) chain	5	6
APC	Adenomatous polyposis coli protein	4	6
CBP	CREB-binding protein	4	6
COOA1	Collagen alpha-1(XXIV) chain	4	6
MYH13	Myosin-13	4	6
RBP2	E3 SUMO-protein ligase RanBP2	4	6
RP1L1	Retinitis pigmentosa 1-like 1 protein	4	6
ANKH1	Ankyrin repeat and KH domain-containing protein 1	3	6
CMYA5	Cardiomyopathy-associated protein 5	3	6
CO6A5	Collagen alpha-5(VI) chain	3	6
GCN1L	Translational activator GCN1	3	6
KMT2C	Histone-lysine N-methyltransferase 2C	3	6
MPDZ	Multiple PDZ domain protein	3	6
PDZD2	PDZ domain-containing protein 2	3	6
TCOF	Treacle protein	3	6
AKP13	A-kinase anchor protein 13	2	6
CKAP5	Cytoskeleton-associated protein 5	2	6
FAT1	Protocadherin Fat 1	2	6
MYH14	Myosin-14	2	6
MYO15	Unconventional myosin-XV	2	6
TRIPB	Thyroid receptor-interacting protein 11	2	6
DYH17	Dynein heavy chain 17, axonemal	1	6

**Figure 3:**
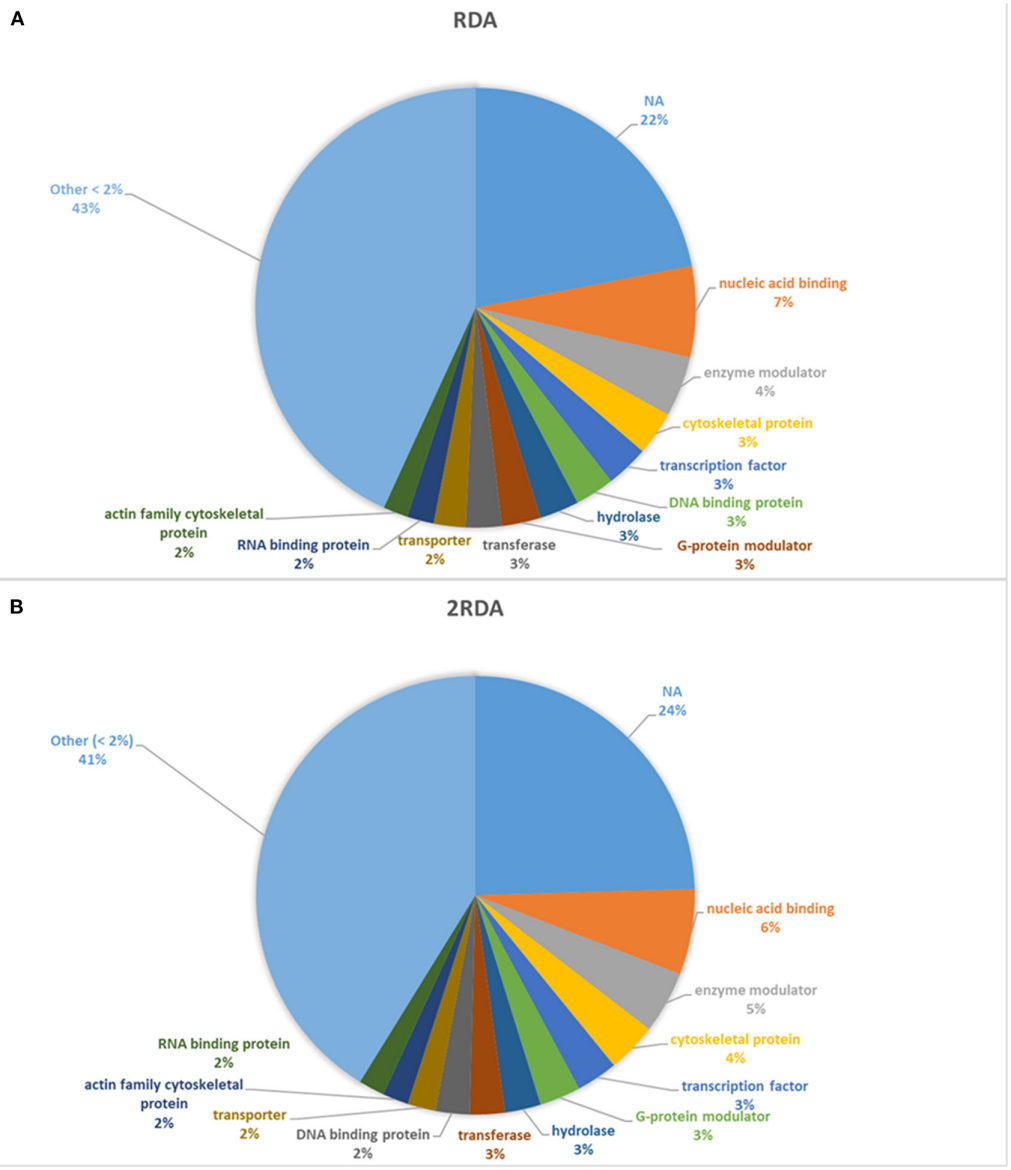
Distribution of protein classes present >2% of reads. NA refers to unassigned proteins. **(A)** includes the 9 participants on the RDA diet and **(B)** refers to the results from the 10 participants in the 2RDA diet.

A diverse number of self-proteins were identified pertaining to a wide array of functions. Many of the proteins had more than one protein function class. In both diets, ~40% of all the identified protein classes were part of a protein class made up of <1% of the total protein. Approximately 20% of the proteins had no class identification according to Panther (see Figure 3). In both diets, the top four classes all had <10% of the total proteins and in descending order were nucleic acid binding, enzyme modulators, cytoskeletal proteins and transcription factors which suggest the presence of shed cells and/or signs of proliferation (nucleic acid binding, cytoskeletal proteins and transcription factors) in faeces. Overall there were limited differences observed in the protein classes between each diet.

The identified self-proteins included those that would be expected to be present in the faeces, including; enzymes (e.g., chymotrypsin), zinc fingers, collagen, myosin proteins, and mucin proteins. In this study, four mucins were identified: MUC4, associated with membranes, in one sample for the RDA diet group; MUC16 (also known as CA125) was found in samples from the RDA diet; MUC5A, a secreted mucin, found in one sample from the 2RDA diet group and MUC19, a secreted protein, which was found in three RDA samples and four 2RDA samples (33, 34). MUC19 helps to maintain the permeability of the intestinal epithelial layer and regulation of immune responses. The presence of this protein may suggest the presence of inflammation or other intestinal disease as the protein has not been previously reported in healthy human intestines (35). All of the participants in this study were healthy but further work could be used to determine if it is a marker for asymptomatic inflammation.

One protein, K167 (Antigen KI-67), was unique to the samples from the RDA diet. K167 is used as a sign of proliferation in research studies including cancer prognosis. It has been found to act as a surfactant that helps to keep mitotic chromosomes apart after release into the cytoplasm (36) which suggests that it may have been a sign of cell proliferation occurring in the RDA diet. Evidence is shown on the Protein Atlas website that this protein is expressed in the gastrointestinal tract including the glandular cells of the colon in a number of individuals of different ages and gender (37, 38).

Protein classes were determined using Panther (39) for all of the identified proteins from each diet. From the proteins found in at least 60% of one of the diets (listed in Figure 2C), 39 of the proteins were classified according to their functions in Panther. The most common function identified were cytoskeletal proteins. Nine out of the 10 proteins were observed in at least 60% of the samples from the 2RDA diet group [including cytoskeleton-associated protein 5 (CKAP5) and talin 1 (TLN1)]. Five of the function groups were related to DNA and included the proteins CREB binding protein (CBP), lysine methyltransferase 2A (KMT2A) and methyltransferase 2C (KMT2C). Both cytoskeletal proteins and DNA related proteins are involved in cell proliferation (40, 41) and suggest that increasing protein intake increases cell proliferation. This plausible effect is supported by similar findings in rat colons (42) but is not supported by the presence of K167, a sign of proliferation, only being found in the RDA group. The lack of K167 suggests that these proteins may be involved in proliferation not identified by the K167 antigen or the increase of cytoskeletal and DNA related proteins did not affect the cell proliferation in this study. Work conducted with a cohort of men which included the cohort in this study showed that some microRNAs were altered in the 2RDA diet and that the 2RDA diet also increased the immune systems post-transcriptional regulation (43). The intestine contains many transporter proteins, and proteins with these functions were also observed in this list of proteins, for example, ryanodine receptor 1 (RYR1) and ryanodine receptor 3 (RYR3).

To give an overview of the number of samples in which each of the proteins were found in the two diets, the top 26 proteins observed in the 2RDA diet (according to frequency observed) are listed in Table 1. Twenty of these proteins are found in the intestinal region according to Protein Atlas (38). These proteins had functions related to digestive enzymes, molecular motors, signalling, and cytoskeleton including placement of organelles. Literature searches resulted in 4 proteins that were newly discovered faecal/intestinal proteins. These were Collagen alpha-1(VII) chain (Uniprot ID CO7A1), Protocadherin Fat 3 (Uniprot ID FAT3), Zinc finger protein 469 (Uniprot ID ZN496), and Dynein heavy chain 8, axonemal (Uniprot ID DYH8). The presence of these proteins also supported our hypothesis that proteins are exfoliated from cells found along the intestinal wall are present in faecal matter. This has been observed previously in colorectal cancer screening although it was less common in the healthy subjects (44).

CO7A1 is a fibril that joins the external epithelia to underling stroma (45). This protein has been shown to be upregulated after addition of TNF-α in cultured fibroblasts (46), which suggests that fibroblasts can produce CO7A1. A layer of fibroblast cells are found under the epithelium in the intestine (47). CO7A1 was observed in the majority of samples, in 6 of the RDA samples and 9 of the 2RDA samples, 90 and 67%, respectively. This suggests that peptides of this protein are commonly found in faecal matter. This may be because the protein is observed close to the external layer of the intestine and may be broken off during normal wear and tear as we hypothesised would happen.

FAT3, in humans, is one of four FAT proteins which are members of the cadherin protein family (48, 49). FAT3 is involved in the interactions with the actin cytoskeleton. There is evidence of FAT3 upregulating β-catenin and proteins downstream of the Wnt signalling pathway (50). This is interesting as two other common proteins BCL9 and MACF1 that were both found 8 times in the 2RDA participants are also part of the Wnt signalling pathway and have previously been observed in the intestinal system (38). Proteins in the Wnt pathway are involved in the development of foetuses and in homeostasis in adults. The process eliminates the degradation pathway leading to an increase of β-catenin both in the cytoplasm and nucleus of cells and increasing transcription and thus protein expression, cell growth and are potentially involved in cell to cell adhesion. In some situations, this function can lead to tumour growth (51) and possibly could be part of the reason that a mutation in FAT3 has been shown to result in pancreatic tumours (50). Since FAT3 is part of the Wnt signalling pathway and other members of this pathway were observed in the samples, it is possible that this pathway was active in the intestinal system which led to fragments of these proteins being found in the faecal samples.

Little is known of the function of ZN469 except that it is a transcription factor like other zinc finger proteins (34). Some research has shown that a peptide from ZN486 is present over twice as much in serous ovarian cancer tissue compared to healthy ovarian epithelium tissue (52). It had been hypothesised that ZN486 polymorphisms could cause keratoconus and reduces vision abilities, but studies from Poland and Saudi Arabia have found that it does not (53, 54). Further research would need to be conducted to theorise and determine the exact function of ZN486 and why it could be present in faecal matter.

DYH8 is an axonemal heavy chain dynein. These heavy chain proteins form a major part of the dynein molecular motors along with minor chain dynein proteins (55, 56). Dynein complexes transport biomolecules along microtubules within cells (56). In staining studies, DYH8 showed a strong presence in testis (38). DYH8 was found in 70% of the 2RDA participants but only 22% of the RDA participants. We suspect that because of the role this protein has in transportation within cells, this could have entered the intestinal system via breakdown of cells.

An additional two proteins that have not been noted from faecal/intestinal proteins, but are found in muscle, were ryanodine receptor 1 (Uniprot ID RYR1) and ryanodine receptor 3 (Uniprot ID RYR3). Ryanodine receptors are part of calcium channels and trigger muscle contraction. There are three of these receptors that are each dominant in different types of muscle. RYR1 is dominant in skeletal muscle whereas RYR3 is dominant in smooth muscle which is found in the intestine. Antibody assays have shown the presence of RYR3 in the intestine (38, 57–59).

Prior to analysis the proteins were digested with trypsin. The cleavage sites of the peptides were examined to see if there were non-tryptic cleavages suggesting breakage of the proteins *in situ*. A peptide was considered to be tryptic if it was the start or end of the protein or a breakage after lysine or arginine. An average of 0.4% of peptides in each sample contained no tryptic cleavages; 8.3% had tryptic cleavages at both ends; and 91% had a tryptic cleavage at one end of the peptide. Overall the average lysine and arginine cleavage sites were 31 and 27%, respectively, in both diets. This suggests that the trypsin digestion accounted for the majority of cleavages but it should be noted that trypsin is used to digest proteins in the small intestine (1) so some of the cleavages may have occurred during this phase of digestion rather than during sample preparation. This work involved extractions optimal to proteins rather than peptides from the faecal samples which suggests the presence of intact host-proteins in the faecal samples that are not completely digested in the small intestine's digestive system.

The participants were older men and hence may not be reflective of the population variation that might exist in women, younger populations or if variation is impacted by age. What was shown is that despite markedly different diets and adherence to a prescribed diet, in this case a high protein diet, there was little or no evidence of increasing uniformity of the proteome in healthy participants. Nor was there a discernible effect of the diet itself.

Thus, analysis and comparison between a healthy and defined unhealthy population in which aspects of GIT function are compromised would be beneficial. Any studies in this area need to be scaled appropriately to take account of the large degree of individual variation in faecal protein composition. The addition of pre-diet samples would help to understand variability prior to the study and add to understanding what self-proteins change with twice the recommended protein allowance. This work was done using a qualitative approach which allows all proteins identified to be studied rather than just those with appropriate quantitative information (60). Further studies using label-free quantitative mass spectrometry could add to the information we have on human proteins by determining differences in the concentration of faecal proteins between the two diets.

## Conclusions

In this study we identified human proteins present in the faeces of elderly men after 10 weeks on a healthy diet consisting of either the RDA or twice the RDA of protein. We used a qualitative approach and found no significant differences between these two healthy diets which suggests that the protein increase used in the 2RDA diet of this study does not affect the shedding of cells and secretion of digestive enzymes into the faecal matter. We did observe that the human host faecal proteome is variable in a set of individuals matched to age and sex. Limited differences in the proteins identified and protein classes was observed between the diets. But there is evidence of proteins relating to cell proliferation more often in the 2RDA than the RDA diet. Higher protein or longer-term diet studies may show further differences not observed in this work. During this process we have been able to show the host-proteome of human faeces from these men. There is no dominant class of proteins, but nucleic acid binding and enzyme modulators are more dominant in the human faeces of these men than other Panther protein classes. Four proteins, CO7A1, FAT3, AN469, and DYH8, previously unidentified in faecal matter were present in at least 70% of the 2RDA diet samples. Since limited differences were observed in the two diets, we now have a foundation to compare to for future human faeces studies. An extension of this foundation that looks at a larger number of participants and especially takes into consideration the inter-individual variation would be beneficial. This extended baseline could be compared to further understand intestinal biopathways or looking for markers of disease. It would be advantageous for future studies to use more extreme variations in dietary habits (omnivore vs. vegan) or from longer intervention studies to see if protein effects are observed when comparing extremes or over extended time frames. Such studies are warranted as GIT dysfunction is a major cause of ill-health, with many people experiencing syndromes for which symptoms are varied and frequently overlapping.

## Data Availability Statement

The datasets generated in this study can be found in online repositories. The names of the repository/repositories and accession number(s) can be found below: PRIDE with identifier PXD021424, http://proteomecentral.proteomexchange.org/cgi/GetDataset?ID=PXD021424.

## Ethics Statement

The studies involving human participants were reviewed and approved by Southern Health and Disability Ethics Committee (New Zealand; 15/STH/236). The patients/participants provided their written informed consent to participate in this study.

## Author Contributions

DC-S, CM, JG, and AG: conceptualisation. DC-S, CM, and AM: dietary methodology. EL, AT, JG, and AG: faecal analysis. CM, AM, SM, NZ, FR, and PS: investigation. JG: data curation and writing—original draught preparation. AM, CM, NZ, FR, PS, SK, NR, AS, K-HW, DC-S, and AG: writing—review and editing. DC-S, NR, SK, AS, and K-HW: funding acquisition. All authors approved the final version of the manuscript for submission.

## Conflict of Interest

The authors declare that the research was conducted in the absence of any commercial or financial relationships that could be construed as a potential conflict of interest.
